# Liquid Chromatographic Determination of Biogenic Amines in Fish Based on Pyrene Sulfonyl Chloride Pre-Column Derivatization

**DOI:** 10.3390/foods9050609

**Published:** 2020-05-09

**Authors:** Elvira S. Plakidi, Niki C. Maragou, Marilena E. Dasenaki, Nikolaos C. Megoulas, Michael A. Koupparis, Nikolaos S. Thomaidis

**Affiliations:** Laboratory of Analytical Chemistry, Department of Chemistry, National and Kapodistrian University of Athens, Panepistimioupolis Zografou, 15771 Athens, Greece; eplakidi@hcmr.gr (E.S.P.); mdasenaki@chem.uoa.gr (M.E.D.); n.megkoulas@qualimetrix.com (N.C.M.); koupparis@chem.uoa.gr (M.A.K.); ntho@chem.uoa.gr (N.S.T.)

**Keywords:** bioamines, polyamines, scombroid poisoning, seafood, dietary exposure, HPLC, pyrene probe, excimer fluorescence, intramolecular excitement

## Abstract

Monitoring of biogenic amines in food is important for quality control, in terms of freshness evaluation and even more for food safety. A novel and cost-effective method was developed and validated for the determination of the main biogenic amines: histamine, putrescine, cadaverine, spermidine and spermine in fish tissues. The method includes extraction of amines with perchloric acid, pre-column derivatization with Pyrene Sulfonyl Chloride (PSCl), extraction of derivatives with toluene, back-dissolution in ACN after evaporation and determination by reversed phase high performance liquid chromatography with UV and intramolecular excimer fluorescence detection. The structure of the pyrene-derivatives was confirmed by liquid chromatography–mass spectrometry with electrospray ionization. The standard addition technique was applied for the quantitation due to significant matrix effect, while the use of 1,7-diaminoheptane as internal standard offered an additional confirmation tool for the identification of the analytes. Method repeatability expressed as %RSD ranged between 7.4–14% for the different amines and recovery ranged from 67% for histamine up to 114% for spermine. The limits of detection ranged between 0.1–1.4 mg kg^−1^ and the limits of quantification between 0.3–4.2 mg kg^−1^. The method was applied to canned fish samples and the concentrations of the individual biogenic amines were below the detection limit up to 40.1 mg kg^−1^, while their sum was within the range 4.1–49.6 mg kg^−1^.

## 1. Introduction

**Figure 1:**
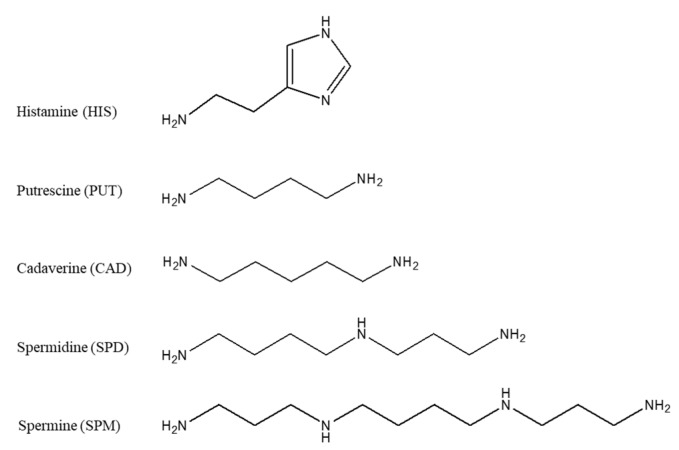
Molecular structures of the biogenic polyamines.

Biogenic amines (BAs) have been widely associated with food quality and safety [1,2,3,4]. Although they are naturally occurring substances in animals and humans, their presence in food is mainly a result of bacterial growth and spoilage through the decarboxylation path of free amino acids [3,4]. Histamine, putrescine, cadaverine, spermidine and spermine (Figure 1), are considered among the most important biogenic amines occurring in food, and they have been used for the generation of a chemical quality index of canned tuna [5] and Mediterranean hake stored in ice [1]. Apart from the effect on the quality of food as regards aspects like the freshness, the presence of BAs in food can endanger food safety, since the dietary exposure of humans to BAs, through consumption of food and beverages with high levels of these compounds, can have serious toxicological effects on human health [4]. The most well-known biogenic amine is histamine, which is present in great abundance in fish and fishery products, and is the main component in “scombroid poisoning” or “histamine poisoning” [2]. Putrescine and cadaverine, are also associated with this illness and their presence has been reported to enhance the toxicity of histamine [2,6]. Moreover, it has been reported that biogenic amines, especially putrescine and cadaverine, may also be considered as carcinogens because of their ability to react with nitrites to form potentially carcinogenic nitrosamines [6,7]. Foods likely to contain high levels of biogenic amines include fish, fish products and fermented foodstuffs (meat, dairy, vegetables, beers and wines) [4].

In order to protect human health, the European Union has established safety criteria for histamine levels in fishery products that range between 100 and 400 mg kg^−1^ [8]. On the other hand, in the United States of America, according to FDA and EPA safety levels, the decomposition criteria limit of histamine for scombrotoxin-forming fish, like tuna, mahi-mahi, and related fish is 50 mg kg^−1^, while the toxic limit of histamine is 500 mg kg^−1^ [9].

According to the scientific opinion on risk-based control of biogenic amine formation in fermented foods published by the European Food Safety Authority (EFSA), no adverse health effects were observed after exposure to 50 mg of histamine in food per person per meal for healthy individuals, but below detectable limits for those with histamine intolerance, while for putrescine and cadaverine, the information was insufficient in that respect [4]. As pointed out in the EFSA scientific opinion, monitoring of BAs’ concentrations in fermented foods during the production process, and along the food chain, would be beneficial for controls and further knowledge. The opinion concludes that additional research is required on several aspects of BAs including validation of the methods of analysis.

Based on the above, it becomes evident that accurate and of high detectability analytical methods for the determination of BAs in food are necessary in order to generate reliable data for a safe estimation of human dietary exposure to biogenic amines. As a result, a number of analytical methods has been developed for the determination of BAs in foodstuff, including capillary electrophoresis (CE) [10,11,12], gas chromatography (GC) with mass spectrometric detection [13,14], ion chromatography with conductimetric and amperometric detection [15] and thin layer chromatography with densitometric scanning [16]. Amongst them, undoubtedly, the most widely applied analytical technique is liquid chromatography (LC) with ultraviolet absorbance (UV) [17,18,19,20,21,22,23,24,25], or fluorescence detector (FLD) [26,27,28,29,30,31,32,33,34,35,36]. The use of mass spectrometric detection is still relatively limited [37,38,39,40].

Due to the lack of chromophore or fluorophore groups in the most of the BAs structures, a pre-column [17,18,19,20,21,22,23,24,25,26,27,28,30,33,34,35,36] or post-column [29,31,32] derivatization step is required. The derivatization reagents tested for the liquid chromatographic determination of BAs include dialdehydes such as o-phthalaldehyde (OPA) [27,29,30,31,32] and naphthalene-2,3-dicarboxaldehyde (NDA) [33], sulfonyl chlorides such as dansyl chloride (Dns-Cl) [17,19,25], dabsyl chloride (Dabs-Cl) [19,25] and 10-ethyl-acridine-3-sulfonyl chloride [19,26], benzoyl chloride [18,19,22,23], succinimidyl reagents like 6-aminoquinolyl-N-hydroxy-succinimidyl carbamate [21], N-(9-fluorenylmethoxycarbonyloxy) succinimide (Fmoc-OSu) [36] and 1-pyrenebutanoic acid succinimidyl ester (PSE) [35], as well as diethyl ethoxymethylenemalonate [20,24].

Among the aforementioned derivatization agents, 1-pyrenebutanoic acid succinimidyl ester (PSE) presents special interest because pyrene derivatives of some biogenic polyamines exhibit intramolecular excimer fluorescence [35]. In case of pyrene derivatives with two or more pyrene moieties and favorable structural conformation, excimer fluorescence is generated by the energy transfer from one pyrene moiety to the other, which results in intramolecular excitement [41]. Excimer fluorescence induces a significant shifting of the emission to higher wavelengths and, therefore, selectivity is increased compared to mono-pyrene derivatives of biogenic monoamines. This derivatization agent has been used for the fluorescence determination of polyamines, putrescine, cadaverine, spermidine and spermine as indicators of food decomposition without chromatographic separation [41], and for their determination in different solvents after liquid chromatography [42]. However, the chromatographic determination of histamine in solvents after derivatization with PSE was not possible [42]. Moreover, when PSE was used for the chromatographic determination of the same polyamines in fish samples, only putrescine and cadaverine were satisfactorily determined [35]. The chromatographic determination of histamine using pyrene reagents for the generation of the excimer fluorescence has been reported to be successful in urine samples with PSE where histamine was the sole analyte [43] and in soy sauce using 2-chloro-4-methoxy-6-(4-(pyren-4-yl)butoxy)-1,3,5-triazine, a derivatization reagent which was laboratory synthesized [44].

**Figure 2:**
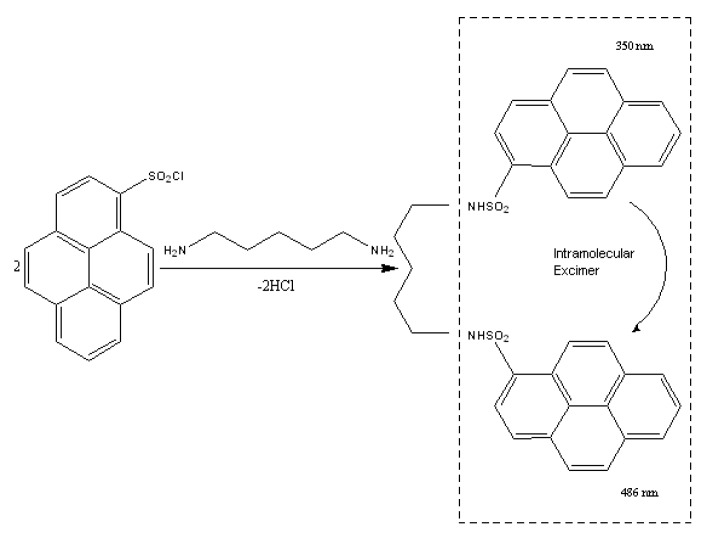
Intramolecular excimer-forming fluorescence derivatization of cadaverine with PSCl.

The present work proposes 1-pyrenesulfonyl chloride (PSCl), as a new derivatization reagent for the simultaneous determination of histamine (HIS), putrescine (PUT), cadaverine (CAD), spermidine (SPD) and spermine (SPM) in fish samples based on fluorescence excimer as presented in Figure 2, and UV detection in series, after high performance liquid chromatography. Pyrene sulphonyl chloride has been used in the past as a reagent for quantitation of estrogens in human serum after liquid chromatography and fluorescence detection [45]. The conditions of the derivatization reaction, including: pH, amount of PSCl, temperature and time, were optimized and the structures of the produced derivatives of the tested polyamines were confirmed by liquid chromatography coupled with electrospray mass spectrometry (LC-ESI-MS). Liquid chromatographic separation and detection wavelengths were also optimized in order to obtain the maximum selectivity and sensitivity. The proposed LC-UV-FLD method was validated and the stability of the PSCl derivatives of BAs was investigated.

To the best of our knowledge this is the first study that presents the simultaneous determination of five significant biogenic amines, including histamine, for the quality and safety control of fish using pyrene sulfonyl chloride in order to induce intramolecular excimer fluorescence. In addition, the present study enlightens some aspects of histamine chromatographic behavior after derivatization with a pyrene reagent, reporting for the first time the existence of three chromatographic peaks which were identified with absorbance and emission spectra, as well as with mass spectrometry, addressing to some extend the reported difficulties of the chromatographic determination of histamine after derivatization with pyrene reagents [35,42].

Comparing to other derivatization agents, like Dns-Cl and OPA, the most significant advantage of the use of PSCl is the increased selectivity towards the biogenic polyamines investigated in the present study, which are derivatized to compounds that exhibit intramolecular excimer fluorescence at high wavelengths, where there is no interference from other endogenous biogenic monoamines or the excess of the derivatization reagent. Moreover, comparing to the derivatization of amines with Dns-Cl [17], the derivatization with PSCl requires much shorter reaction time, 15 min versus 20 h, and offers the possibility of the use of both detectors UV and fluorescence, increasing the degree of analyte confirmation, whereas Dns-Cl derivatized amines are detected only with UV. Finally, the smaller amount of sample and organic solvents used for the sample preparation of the present study (2 g sample, 300 μL toluene and 300 μL acetonitrile) comparing to studies using Dns-Cl (40 g sample, 3 mL heptane and 1.5 mL acetonitrile) [17] and OPA (50 g sample, 6 mL ethyl acetate and 1 mL acetonitrile) [27] for the derivatization of BAs in fish, as well as the use of a low-cost readily available HPLC-UV/FLD instrumentation comparing to mass spectrometry instruments [38,39], render the present method rather cost-effective and eco-friendly.

## 2. Materials and Methods

### 2.1. Standards and Reagents

HPLC-grade water (specific resistance > 17.8 MΩ cm) was obtained by a Milli-Q water purification system (Millipore, Billerica, MA, USA), acetonitrile (ACN) of HPLC grade was purchased from LAB SCAN (Dublin, Ireland) and toluene from Mallinckrodt (Surrey, UK). The derivatization reagent Pyrene Sulfonyl Chloride (PSCl, C_16_H_9_ClO_2_S, MW: 308 g mol^−1^) was obtained from Molecular Probes (Eugene, Oregon, OR, USA). A 3.0 mM PSCl solution was prepared by dissolution of 45 mg of the reagent in 50 mL ACN and was stored at −20 °C for 6 months. For the extraction procedure, an aqueous solution of 0.2 M perchloric acid (HClO_4_, 70–72% reagent grade, Merck, Whitehouse Station, New Jersey, NJ, USA) and a saturated sodium carbonate (Na_2_CO_3_, purity ≥ 99.5%, Merck) solution in water were prepared. 

The hydrochloric salts of the biogenic amines: (a) histamine dihydrochloride (99%, Sigma-H7250), (b) putrescine dihydrochloride (98%, Sigma-P7505), (c) cadaverine dihydrochloride (98%, Sigma-C8561), (d) spermidine trihydrochloride (98%, Sigma-S2501) and (e) spermine tetrahydrochloride (Sigma-S2876), as well as 1,7-diaminoheptane dihydrochloride (98%, Aldrich-D 17408), used as internal standard, and L-proline (99.0%, Fluka-81710) were purchased from Sigma-Aldrich (St. Louis, Missouri, USA). Individual standard stock solutions were prepared by dissolution of the appropriate hydrochloric salt equivalent to 10.0 mg of each amine in 100.0 mL of water and were stored at 4 °C for one month. Working standards solutions of mixtures of the BAs were prepared by appropriate dilutions of the stock solutions with 0.2 M HClO_4_. A 2% *w/v* L-proline aqueous solution was also prepared, used as quenching agent of the derivatization.

### 2.2. Instrumentation

A centrifuge equipment (Rotofix 32-Hettich, Merck, Darmstadt, Germany), a pH meter (Metrohm, Herisau, Switzerland) and a thermostated evaporation aluminum block under nitrogen flow were used for the sample treatment.

HPLC-UV-FLD analysis was carried out on an Agilent 1100 LC modular system (Agilent Technologies, Wilmington, DE, USA) consisting of a G1311A quant pump, a G1379A degasser, a G1321A fluorescence detector (FLD), a G1314A ultra-violet detector (UV) and a 7725i Rheodyne (California-USA) manual sample injector equipped with a 20 μL loop.

The structural elucidation of PSCl derivatives was performed with a Thermo Finnigan LC–MS system (San Jose-CA) consisted of a Spectra System P 4000 pump, a Spectra System AS 3000 autosampler with the volume injection set to 20 μL and a MSQ quadrupole mass spectrometer equipped with an electrospray ionization interface (ESI).

### 2.3. Fish Samples

Six kinds of canned fish including (a) sea bass, (b) anchovy, (c) anchovy marinated, (d) mackerel smoked, (e) sardines in oil and (f) tuna in water and salt, were obtained from local markets in Athens. The whole content of each can was homogenized with a laboratory homogenizer and stored at −15 °C. Aliquots of the homogenized samples were used for method optimization and validation. All the samples in total were measured according to the optimum protocol for the determination of biogenic amines content.

### 2.4. Method Development

#### 2.4.1. Extraction of Biogenic Amines

Aliquots of 2 g of fish homogenate, accurately weighted, were placed in a 15 mL plastic centrifuge tube and spiked with 2.4 μg of internal standard (24 μL of 100 μg mL^−1^ standard solution). Eight mL of 0.2 M perchloric solution were added, the tube was capped and vortexed for 5 min. Afterwards, the tube was centrifuged at 4000 rpm for 15 min and the supernatant layer was isolated for the derivatization of biogenic amines. 

#### 2.4.2. Optimization of Derivatization of Biogenic Amines–Final Protocol

The effect of pH, temperature, reaction time and amount of derivatization reagent on the derivatization yield was investigated using a standard mixture containing 1 mg L^−1^ of the tested biogenic amines and 0.3 mg L^−1^ of the internal standard and also with anchovy sample spiked with biogenic amines at a measured concentration of 1 mg L^−1^. The optimization was performed according to the univariant technique and the effect of the tested parameters was evaluated based on the peak area of the FLD chromatograms. 

*Study of pH:* Since the pyrene sulfonyl chloride derivatization reaction with the biogenic amines is a nucleophilic substitution, a moderate alkaline medium is required so that the biogenic amines become deprotonated. However, a highly alkaline medium must be avoided, since a successive quick decomposition by alkaline hydrolysis of the produced pyrene derivatives may be observed. The pH values tested ranged between 7 and 11, adjusted with the addition of different volumes of a saturated sodium carbonate solution. A volume of 400 μL of 3 mM PSCl solution was used and the derivatization took place at 60 °C for 15 min.

*Study of temperature and time:* The tested temperatures for the derivatization were 22, 30, 40, 50, 60, 70 and 80 °C with a reaction time of 15 min. The optimization of the derivatization temperature was conducted at pH of 9.6 with the addition of 30 μL of saturated sodium carbonate solution and 400 μL of 3 mM PSCl solution. Afterwards, the derivatization time at the optimum temperature was examined for a duration between 15 and 60 min.

*Study of derivatization reagent amount:* For the determination of the optimum amount of the derivatization reagent, different volumes between 200–1200 μL of 3 mM PSCl solution in ACN were tested. The pH was set at 9.6 with the addition of 30 μL of saturated sodium carbonate solution and the derivatization was left to take place at 60 °C for 15 min.

*Final protocol of derivatization*: First, 300 μL of the supernatant layer, which was isolated during the extraction step (Section 2.4.1), was transferred into a 10 mL glass tube, wrapped with aluminium foil to protect the derivatives from light. Subsequently, 30 μL of saturated sodium carbonate solution was added in order to set the pH at 9.6. Then, 600 μL of 3.0 mM solution of the derivatization reagent PSCl was added, the tube was capped, vortexed and heated in a water bath at 60 °C temperature for 15 min. After being cooled down at room temperature, 100 μL of a 2% *w/v* L-proline aqueous solution was added to stop the derivatization reaction. The tube was left for 15 min at ambient temperature. Derivatives were extracted from the aqueous phase by liquid-liquid extraction with 300 μL of toluene, followed by centrifugation at 3000 rpm for 10 min. The supernatant organic phase was collected into an Eppendorf tube and toluene was evaporated to dryness under a gentle nitrogen stream. The dry residue was reconstituted in 300 μL ACN and then filtered through a 0.2 μm syringe filter prior to LC injection.

#### 2.4.3. HPLC-UV-FLD Analysis

Chromatography was performed on an Agilent Zorbax Eclipse XDB-C18 (150 mm × 4.6 mm, 5 μm) analytical column. Gradient elution of mobile phase consisting of water and acetonitrile was optimized in order to achieve satisfactory chromatographic separation, within the minimum possible analysis time, taking into consideration the interferences of the complex matrix of fish samples. The optimum and finally selected linear gradient elution program started with 40% ACN which increased linearly to 80% up to 25th min and further increased to 100% up to 31st min, afterwards the percentage of ACN decreased to the initial value of 40% up to 42nd min and remained constant for five more min for equilibration of the column, reaching a total analysis time of 47 min. The mobile phase flow rate was 1.2 mL min^−1^ and the column temperature was set at 30 °C.

For the selection of the optimum wavelengths, the emission spectra with constant the excitation wavelength (λ_exc_) and the excitation spectra with constant emission wavelength (λ_em_) for all the pyrene-derivatized amines were recorded. The final selected FLD excitation wavelength (λ_exc_) was 350 nm and the final selected emission wavelengths were 489 nm for PUT (until 23.5 min of the chromatographic analysis), 486 nm for CAD, HIS and internal standard (until 29 min), 484 nm for SPD (until 31 min) and 486 nm SPM (until the end of chromatographic analysis). The UV absorption wavelength was set at 350 nm. 

#### 2.4.4. LC-MS Analysis

For the LC-MS analysis the chromatographic conditions were as follows: LiChrospher 100-RP18 analytical column (250 × 4.0 mm, 5 μm particle size) and isocratic elution with a mobile phase consisting of ACN-H_2_O 80:20 *v/v*, at 1.0 mL min^−1^ flow rate was applied for the analysis of the derivatives of all the tested BAs, except for the analysis of the derivative of SPM, where a mobile phase of 100% ACN was used. ESI was applied in the positive ionization mode and the capillary was held at a potential of 3.5 kV. The cone voltage was 20 V and the ionization source was set at a temperature of 350 °C. A standard solution of 5.0 μg mL^−1^ was prepared for each BA which was derivatized according to the proposed protocol. The full scan spectrum (*m/z* 150–1500) and the total ion chromatogram for each pyrene derivative were acquired. The reconstructed ion chromatograms (RICs) of the most abundant *m*/*z* ions of the spectrum at the retention time of the eluted peak, which could be attributed to specific species of the tested analyte were generated.

### 2.5. Method Validation

The optimized method was evaluated using standard solutions of derivatized BAs and spiked samples of sea bass, anchovy, anchovy marinated, mackerel, sardines and tuna.

The linearity of the derivatization and the response of the HPLC–UV–FLD system was examined with a calibration curve, obtained by triplicate analysis of seven standard solutions in the range of 0.1 to 10.0 μg mL^−1^, which were treated according to the proposed derivatization procedure described in Section 2.4.1 and Section 2.4.2, using 300 μL of the BAs standard solutions of the different concentrations instead of fish sample. The standard solutions contained 0.3 μg mL^−1^ of the internal standard. Linear regression analysis was performed using: (a) the area and (b) the ratio *analyte peak area/internal standard peak area* against analyte concentration and the effect of the use of the internal standard was investigated.

Matrix matched calibration curves were prepared with different fish tissues (sea bass, anchovy, anchovy marinated, mackerel, sardines and tuna). Each sample was spiked at seven fortification levels with the BAs, within the range of 0.2–32 mg kg^−1^. Spiked samples and non-spiked samples of the same batch were analyzed according to the protocol described in Section 2.4.1 and Section 2.4.2. The final linear equation of the matrix matched calibration curve resulted after the subtraction of the signal of the unfortified sample from the signal of the fortified samples.

The instrumental limit of detection (LOD) and quantification (LOQ) were defined as (3.3 × S_a_)/b and as (10 × S_a_)/b, respectively, where S_a_ stands for the standard deviation of the intercept of a low level calibration curve (0.025–0.8 μg mL^−1^) and b for the slope of the same calibration curve. The overall LOD and LOQ of the method were determined applying the same mathematical equations to the matrix matched calibration curves.

The instrumental repeatability of the HPLC-UV-FLD analysis was estimated with five replicates of a standard solution containing 0.5 μg mL^−1^ of the derivatized BAs and 0.3 μg mL^−1^ of the internal standard. For the assessment of the overall precision, the method was applied to an anchovy sample which contained the BAs at a concentration range of 2–10 mg kg^−1^. Sample preparation and HPLC-UV-FLD measurement of six replicates during one day were conducted for the determination of the repeatability (*n* = 6, intra-day precision), and six replicates in two different days, were conducted to test for the intra-laboratory reproducibility of the method (*n* = 6 k = 2, inter-day precision).

For the assessment of the accuracy, the method was applied to a sea bass sample that was spiked with BAs at six fortification levels (1.6, 3.2, 8.0, 12.8, 16.0 and 32.0 mg kg^−1^) and analyzed in triplicate. The absolute recovery (%*R_abs_*) of the method was calculated by dividing the slope of the sea bass matrix matched curve (*b_MM_*) with the slope of the standard curve (*b_STD_*), according to Equation (1). The relative recovery (%*R_rel_*) was calculated by subtracting the concentration measured in the non-spiked sample from that measured in the spiked sample and then dividing with the spiked concentration (*C_ADDED_*) according to Equation (2). The concentrations were calculated from the matrix matched calibration curve of sea bass.

(1) $$\%R_{abs}=\frac{b_{MM}}{b_{STD}}\times 100$$

(2) $$\%R_{rel}\;=\;\;\frac{C_{SPIKED\;SAMPLE}-C_{NONSPIKED\;SAMPLE}}{C_{ADDED}}\times 100$$

## 3. Results and Discussion

### 3.1. Liquid Chromatographic Separation

**Figure 3:**
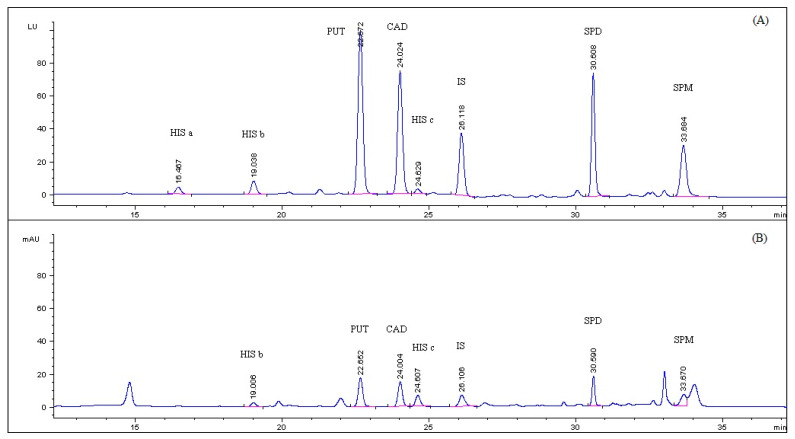
(**A**) HPLC-FLD; (**B**) HPLC-UV chromatogram of PSCl derivatized BAs standard solution of 0.5 μg mL^−1^ and 1,7 diaminoheptane at 0.3 μg mL^−1^. (HIS a, HIS b, HIS c: Histamine, PUT: Putrescine, CAD: Cadaverine, SPD: Spermidine, SPM: Spermine, IS: Internal standard).

**Figure 4:**
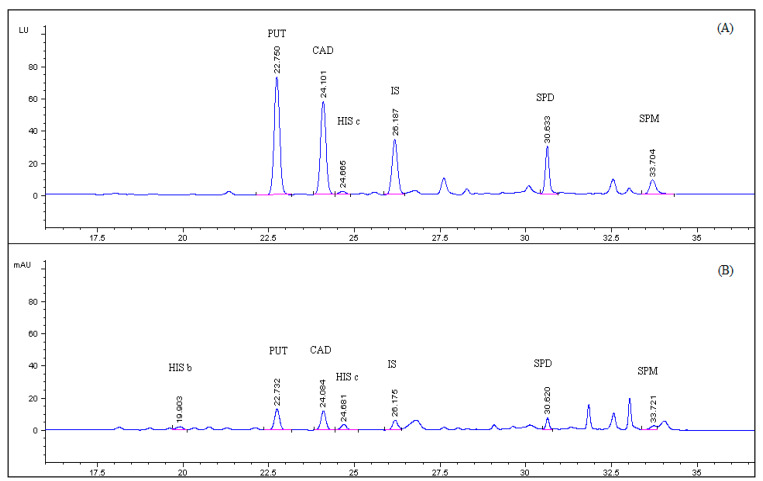
(**A**) HPLC-FLD; (**B**) HPLC-UV chromatogram of spiked sea bass tissue, fortified with putrescine (PUT), cadaverine (CAD), histamine (HIS), spermidine (SPD), spermine (SPM) at 8.0 mg kg^−1^ and 1,7 diaminoheptane (IS) at 4.8 mg kg^−1^.

Representative HPLC-UV and HPLC-FLD chromatograms of PSCl derivatized BAs standard solution of 0.5 μg mL^−1^ and of spiked sea bass blank sample are depicted in Figure 3 and Figure 4, respectively. It was noted that derivatized histamine solution presented three chromatographic peaks (HIS a, HIS b, HIS c) at three retention times at approximately 16.5, 19 and 24.6 min, respectively, in the fluorescence chromatogram and two of them, HIS b and HIS c, in the UV chromatogram. The three eluted peaks of histamine exhibited the same excitation and emission spectra and the same mass spectrometric spectra which correspond to the dipyrene derivatives of histamine (Section 3.2 and Section 3.5). Based on these data, it was hypothesized that the three chromatographic peaks correspond to three isomers of the pyrene derivatized histamine which are generated because of the tautomerism of the imidazole ring. The sum of the peak areas of the three chromatographic peaks were considered for all the calculations for histamine. Representative HPLC-UV and HPLC-FLD chromatograms of an unfortified sea bass sample is presented in Supplementary Material (Figure S1).

The chromatographic parameters, retention time, peak width at 50% height, peak shape in terms of asymmetry factor, chromatographic efficiency in terms of theoretical plates and resolution of all the amines’ pyrene derivatives peaks of a spiked sea bass sample with fluorescence detection are presented in Table 1. Asymmetry factor is in the acceptable range of 0.8–1.2 and resolution is higher than 1.5 in all cases.

### 3.2. Selection of Excitation and Emission Wavelengths

**Figure 5:**
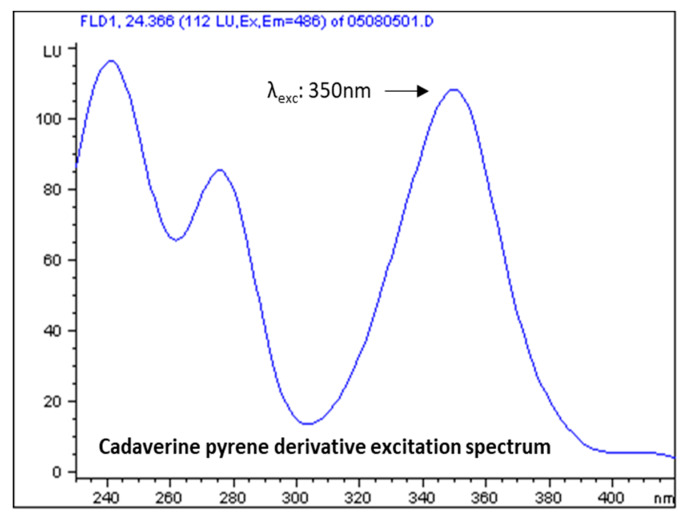
Excitation spectrum of cadaverine pyrene derivative chromatographic peak. Retention time approximately 24 min.

Figure 5 illustrates the excitation spectra of a standard solution of cadaverine derivative at the respective retention time. Similar excitation spectra were obtained for all the pyrene derivatives of the five tested biogenic amines and the internal standard with three peaks at approximately 245, 275 and 350 nm. The excitation wavelength 350 nm was selected for all the analytes.

**Figure 6:**
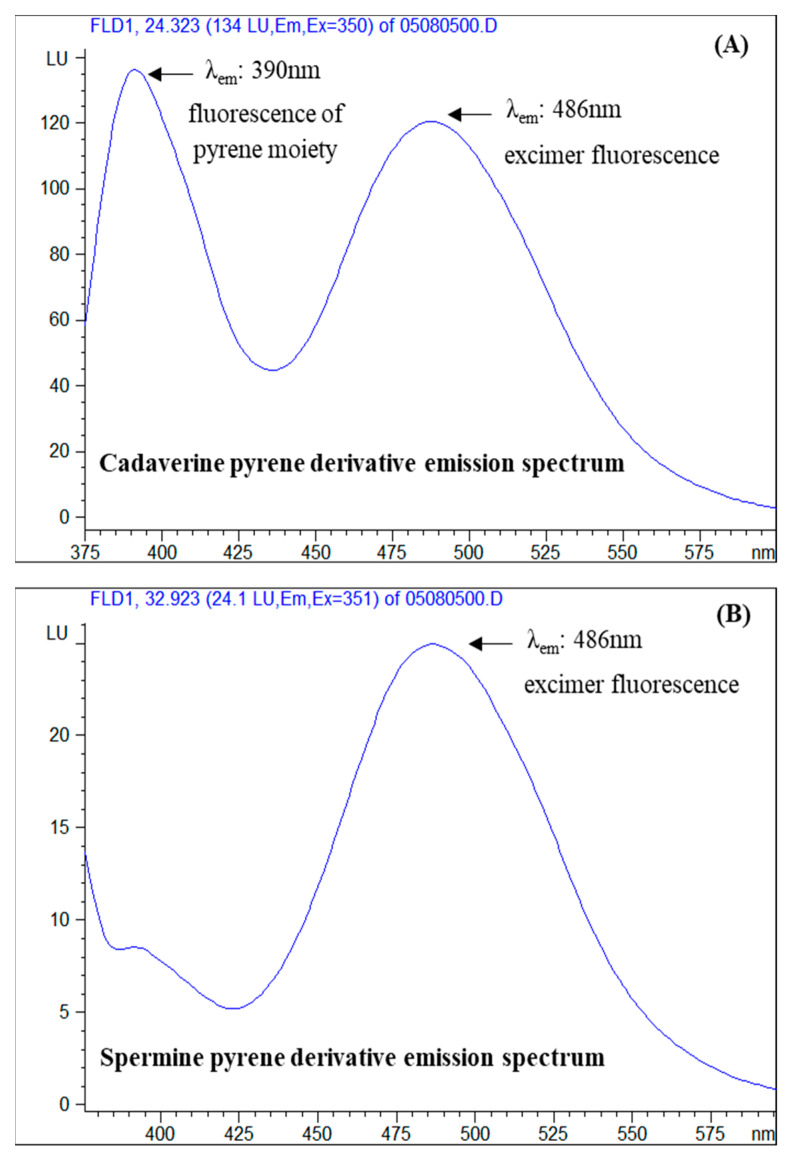
Emission spectra of (**A**) cadaverine and (**B**) spermine pyrene derivative chromatographic peaks. Retention times approximately 24 and 33 min, respectively.

Figure 6 illustrates the emission spectra of a standard solution of cadaverine derivative (A) and spermine derivative (B) at the respective retention times of their eluted peaks. It is observed that the emission spectra of the cadaverine derivative consist of two peaks at approximately 390 nm and 486 nm, whereas in the spermine derivative emission spectrum only the second peak is present. The peak at 390 nm is attributed to the direct fluorescence of pyrene moiety, whereas the second peak which appears at significantly higher wavelengths (486 nm), is considered to be a result of the excimer fluorescence formation. Putrescine, histamine and the internal standard derivatives produced similar emission spectra with cadaverine derivatives with slight variations at the wavelength of maximum excitation, whereas the spermidine derivative emission spectra resembled that of the spermine derivative. The final selected excitation wavelengths are summarized in Section 2.4.3. The emission spectra of the PSCl-derivatized BAs of the present study, with excimer fluorescence peak at 484–489 nm are comparable with the corresponding emission spectra of the PSE-derivatized BAs with excimer fluorescence peak at 475 nm [42].

The excimer fluorescence process is based on the neighboring of the pyrene moieties in a compound, which enables the efficient intramolecular energy transfer from one pyrene moiety to the neighboring one (intramolecular excitement). The energy transfer results in the enhancement of the fluorescence intensity and in the shifting at higher wavelengths. The most critical parameters of the excimer fluorescence are the distance and the conformation between the pyrene moieties, which can favor the intramolecular energy transfer, as for example in the case of cadaverine which is presented in Figure 2. Based on the obtained excitation spectra, the pyrene derivatives of all the tested biogenic amines presented excimer fluorescence, but among them, cadaverine and putrescine derivatives produced the higher fluorescence response factor, which is attributed to their molecular structure (Figure 1).

It is noted that the monitoring emission wavelength was selected to be the wavelength at the maximum emission of the excimer (second peak of emission spectrum) because at this wavelength no interference of the excess of the pyrene derivatization reagent is present, since it does not form fluorescence excimer, and moreover, considerably higher sensitivity for spermidine and spermine at excimer wavelength (484–486 nm) was obtained in comparison to the wavelength of the maximum emission of the pyrene moiety at 390 nm, as can be observed in the spectrum of spermine in Figure 6B.

### 3.3. Optimization of the Derivatization Procedure

#### 3.3.1. Effect of pH

**Figure 7:**
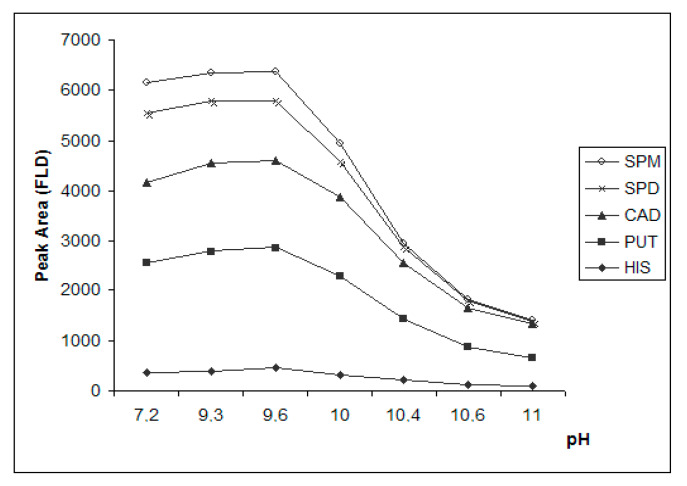
Effect of pH on the efficiency of the derivatization reaction of putrescine (PUT), cadaverine (CAD), histamine (HIS), spermidine (SPD) and spermine (SPM) in standard solution of 1 μg mL^−1^ with 400 μL of 3 mM PSCl.

The effect of the pH on the completion of the derivatization reaction was studied in standard solution of biogenic amines and in spiked anchovy sample extract. Figure 7 presents the effect of pH on the peak area of the different pyrene derivatives of the biogenic amines of a standard solution. It is observed that the optimum pH was 9.6, which was achieved with the addition of 30 μL of saturated sodium carbonate solution to sample extract. At higher pH values the peak area of all the derivatized amines was reduced. It is noted that the optimum pH (9.6) is ≤ than the pKa of the tested amines (HIS-pKa_1_:9.7 [46], CAD-pKa_1_:10.2, pKa_2_:9.1 [47], PUT-pKa:10.8 [48], SPD-pKa:10.9 [49], SPM-pKa:11.1 [50]), except for the imidazole amino group of histamine (pKa_2_:6.0) [46]. As already mentioned, the pyrene sulfonyl chloride derivatization reaction with the biogenic amines is a nucleophilic substitution and amines need to be in their basic form in order to act as nucleophiles. However, at pH ≥ 10 the signal of the produced pyrene derivatives is significantly decreased potentially because of their subsequent alkaline hydrolysis. The same pattern of the peak area change of the derivatized amines in relation to pH was observed in the spiked anchovy sample as well. It is noted that the optimum pH for the derivatization of the BAs under investigation with PSCl is inside the reported pH target range for a robust reaction of putrescine and cadaverine with another pyrene reagent, PSE, which was quite wide, between pH 8 and 11 [35].

#### 3.3.2. Effect of Temperature and Time

**Figure 8:**
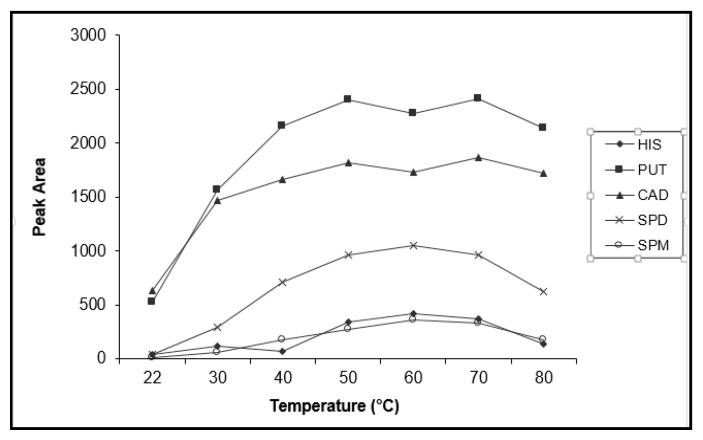
Effect of temperature on the efficiency of the derivatization reaction of putrescine (PUT), cadaverine (CAD), histamine (HIS), spermidine (SPD) and spermine (SPM) in standard solution of 1 μg mL^−1^ with 400 μL of 3 mM PSCl.

Figure 8 presents the effect of reaction temperature on the obtained FLD signal of the derivatized amines. The optimum temperature for histamine, spermidine and spermine derivatization appears to be 60 °C, while for putrescine and cadaverine an optimum plateau is observed between 50–70 °C. Temperatures below 50 °C resulted in lower fluorescence signal, probably due to incomplete derivatization of all the tested amines, and at temperatures above 70 °C partial decomposition of the derivatization product is possibly taking place. The study of the derivatization time showed that incubation for a time period longer than 15 min does not increase the efficiency of the reaction. The optimum derivatization temperature and time required for the derivatization of these polyamines with PSCl (60 °C, 15 min) are very close to the optimum conditions applied for the derivatization of the same amines with other pyrene reagents like PSE (55 °C, 15 min) [35] and 2-chloro-4-methoxy-6-(4-(pyren-4-yl)butoxy)-1,3,5-triazine (50 °C, 20 min) [44]. However, more extreme conditions have also been reported for the same derivatization reaction with PSE (100 °C, 20 min) [42] and (100 °C, 90 min) [43].

#### 3.3.3. Effect of Derivatization Reagent Amount

**Figure 9:**
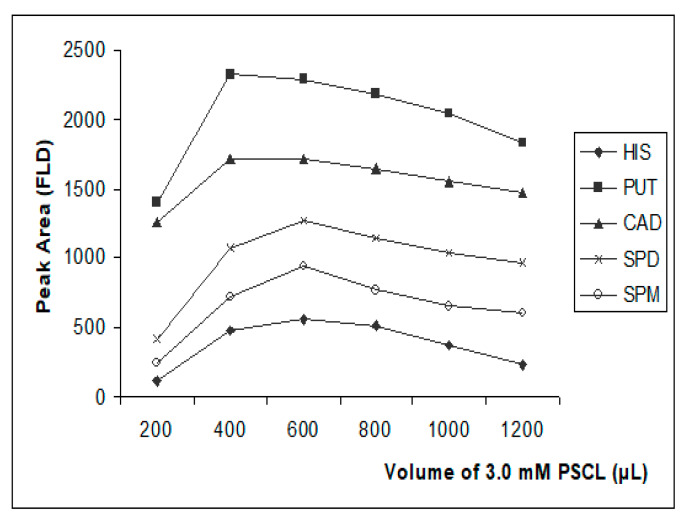
Effect of the volume of the 3.0 mM PSCl solution on the efficiency of the derivatization reaction with putrescine (PUT), cadaverine (CAD), histamine (HIS), spermidine (SPD) and spermine (SPM) in standard solution of 1 μg mL^−1^.

Based on the data presented in Figure 9, a volume of 600 μL of 3.0 mM PSCl solution was selected as the optimum amount for the derivatization of 300 μL of 1 μg mL^−1^ of the five biogenic amines plus the internal standard at 0.3 μg mL^−1^, taking into consideration that histamine, spermidine and spermine, exhibited the maximum sensitivity at this volume and at the same time sensitivity of putrescine and cadaverine was satisfactory. The addition of higher volumes, resulted in a slight decrease of the fluorescence signal for all the analytes which could be attributed to possible quenching of the excimer fluorescence. It is noted that the tested volumes of 3 mM PSCl solution, 200–1200 μL, correspond to 600–3600 nmol of the derivatization reagent which is stoichiometrically in excess, comparing to the required moles, approximately 5 nmol, for the complete derivatization of all the amino groups of the five biogenic amines plus the internal standard, which are contained in the 300 μL of the 1 μg mL^−1^ standard solution.

### 3.4. Stability of the Derivatives

A comprehensive stability study was conducted for a two-month period. A standard solution containing 0.5 μg mL^−1^ of each amine was treated according to the optimum derivatization procedure and stored at 4 °C. Fourteen determinations were performed through the storage time. The variations expressed as %RSD (*n* = 14) of the derivatives’ peak areas through this period ranged from 3.0% for cadaverine up to 15% for spermine. In addition, for all the pyrene derivatives, diagrams of the peak area against time were generated and the slope of these curves was found to be statistically equal to zero (*t*-test, at 95% confidence level), indicating that none of the derivatives presented significant decomposition through the two-month period.

### 3.5. LC-MS Identification of Biogenic Amine Derivatives

Table 2 summarizes the identified *m/z* ions of the tested solutions of the biogenic amines after derivatization with pyrene sulfonyl chloride. The derivatized solution of histamine presented three chromatographic peaks during the LC-MS analysis as observed in Figure S2 (Supplementary Material), similarly to the HPLC-UV-FLD analysis (Section 3.1), in contrast to all the other tested biogenic amines, whose derivatives presented one eluted peak in all the chromatographic analysis. The dipyrene-derivatives of histamine were identified with two *m/z* ions, the protonated molecular ion [M + H]^+^ and the sodium adduct of the molecular ion [M + Na]^+^.

The dipyrene-derivatives of putrescine, cadaverine and internal standard 1,7-diaminoheptane were identified with three *m/z* ions, the protonated molecular ion [M + H]^+^, the sodium adduct of the molecular ion [M + Na]^+^ and the sodium adduct of the dimeric form of the dipyrene-derivatives [2M + Na]^+^. The monopyrene-derivatives were not detected. A representative full scan spectrum of the derivatized cadaverine is depicted in Figure S3, and the corresponding reconstructed ion chromatograms of the three *m/z* ions used for identification are presented in Figure S4 in Supplementary Material.

Accordingly, the tripyrene-derivative of spermidine was identified by the sodium adduct of the molecular ion, whereas no characteristic ions were identified for its mono- or dipyrene-derivatives. Finally, the tetrapyrene-derivative of spermine was identified by the protonated molecular ion. The sodium adduct was not observed in the LC-MS analysis of spermine derivative, since the mobile phase was purely organic (100% ACN). No characteristic *m/z* ions could be identified for the mono-, di- or tripyrene-derivatives of spermine. Based on the above, it can be concluded that all the primary and secondary amino groups of the investigated amines were completely derivatized under the optimum conditions.

### 3.6. Method Performance

Table 3 shows the equations of the calibration curves prepared from the analysis of standard solutions of the investigated derivatized amines using as analytical parameter the peak area. The standard deviation of the slope and the intercept, and the correlation coefficient of each equation are also given along with the instrumental limit of detection (LOD_instr_) and quantification (LOQ_instr_). 

Table 4 presents the corresponding information of the calibration curves prepared from the analysis of spiked sea bass sample, as well as the method limit of detection (LOD) and quantification (LOQ) for both detectors. It is demonstrated that the overall method, and therefore the derivatization procedure, presented linearity for the tested concentration range with correlation coefficients always exceeding 0.992 for both detectors. Representative matrix matched calibration graphs of sea bass are presented in Supplementary Material (Figure S5).

The data of Table 3 and Table 4 show that the FLD slopes of the calibration curves are five to six times higher than the corresponding slopes of the UV detector for the standard and sample solutions for all the derivatized BAs, except for histamine which exhibited similar sensitivity for both detectors. Among the tested BAs, the higher sensitivity was obtained for putrescine and cadaverine, probably due to their stereochemical conformation, which favored the fluorescence excimer process.

Significant matrix effect was observed for all the fish samples (sea bass, anchovy, anchovy marinated, mackerel, sardines and tuna), since t-test analysis at 95% confidence level showed that the slopes of the standard calibration curves differ significantly from the corresponding slopes of the matrix matched curves. In order to address the matrix effect, internal standardization was investigated. Compound 1,7-diaminoheptane was selected as a suitable internal standard, since its structure is similar to the linear polyamines tested, in particular to putrescine and cadaverine. Linear regression analysis was performed on the data obtained from the standard solutions and the spiked samples using the ratio analyte peak area/internal standard peak area against analyte concentration. Despite the satisfactory correlation coefficients (r > 0.99), the slopes of the matrix matched curves were still statistically different from the corresponding slopes of the standard calibration curves, indicating that the use of the internal standard could not compensate for the matrix interferences. Based on these findings it was concluded that for the quantification of the tested BAs in fish samples standard addition is necessary.

The LODs of the method for all the tested matrices for the five BAs are presented in Table 5 and they range between 0.1 to 1.4 mg kg^−1^ for both detectors, UV and FLD. The corresponding method LOQs range between 0.3–4.2 mg kg^−1^. It is noted that similar limits of detection and quantification were obtained for both detectors, UV and FLD, with small variation among the different biogenic amines (Table 3, Table 4 and Table 5). The LODs and LOQs of the proposed method are adequate for the safety control of food products, since they are much lower than the existing regulated limits of histamine, which range between 100–500 mg kg^−1^ [8,9]. In addition, they are comparable to the LODs and LOQs of other reported analytical methods of the same scope [17,26,27,33,34,35].

The instrumental repeatability, the intra- and inter-day method precision data are given in Table 6. For the evaluation of the precision of the proposed method, external standardization technique was compared to internal standardization with the use of 1,7-diaminoheptane. The repeatability of liquid chromatographic peak areas (PA) of the linear biogenic amines (putrescine, cadaverine, spermidine and spermine) PSCl derivatives ranges between 1.8–2.3% for UV detection and between 2.6–2.8% for fluorescence detection. Chromatographic repeatability is considerably improved by internal standardization and the use of ratio F, decreasing to 0.1–0.7% for both detectors. As already mentioned, standard solution of PSCl derivatized histamine exhibits a different chromatographic behaviour with three chromatographic peaks, attributed to the PSCl derivatives of three isomeric structures of the molecule. The higher %RSD values of 4.2% for UV and FLD of the peak area of derivatized histamine are potentially related to the presence of these isomers. A slight improvement by the use of the internal standard and the ratio F is observed in case of histamine.

According to Table 6 overall method repeatability (intra-day assay) ranges between 8.2–13% for UV and 7.4–14 for FLD and overall method reproducibility (inter-day assay) ranges between 8.9–14% for UV and 9.6–21 for FLD using the peak areas of the analytes as analytical parameters, while no significant improvement is gained with the use of the internal standard, ratio F. Based on these data, it can be concluded that the UV signal is slightly more robust compared to FLD and that the use of internal standard cannot compensate for the variation of the signal attributed to the complex matrix. However, internal standard can serve as an additional confirmation tool for the identification of the presence of the biogenic amines in an unknown sample. Two-month stability data (*n* = 14) on the retention time of the derivatized amines and their relative retention time (RT_BA_/RT_I.S._) revealed that the %RSD of the retention times ranged between 0.16–0.37% while the relative retention times ranged between 0.04–0.22.

The accuracy of the developed method expressed as absolute and relative recovery % of the biogenic amines is presented in Table 7. It is noted that for the first three eluted peaks of the derivatized putrescine, cadaverine and histamine detected by UV the absolute recovery ranges between 66% and 75% whereas it is remarkably decreased for the two last eluted peaks of the derivatized spermidine (27%) and spermine (12%). Similar results are obtained with fluorescence detector. The effect of the standard addition on the quantification of the biogenic amines in fish samples and the subsequent improvement on the accuracy of the method is described by the increased values of the relative recoveries for UV (72–112%) and FLD (67–114%). 

### 3.7. Concentration Levels of Biogenic Amines in Fish Samples

Table 8 presents the mean concentration of the biogenic amines, putrescine, cadaverine, spermidine, spermine and histamine, that was determined in commercial canned fish samples and their sum. All the five biogenic amines tested were detected in mackerel, anchovy and sardine samples. In tuna sample no putrescine and histamine was detected and in sea bass sample no cadaverine and histamine was detected. The highest concentration is observed for putrescine in mackerel at 40.1 mg kg^−1^ and the highest sum of biogenic amines is observed for mackerel and sardines at 49.6 and 34.9 mg kg^−1^, respectively. It is noted that all results are below the strictest EU limit for histamine of 100 mg kg^−1^.

These findings are within the range of other reported surveys on these biogenic amines’ levels in similar fish products. In particular, according to a European survey the reported mean concentrations of histamine in fish and fish products range between 29.3–33.6 mg kg^−1^, with ‘dried anchovies’ to be the subgroup of fish products with highest mean values of histamine, at 348 mg kg^−1^ [4]. According to a recent study in Cambodian fermented foods, histamine in fishery products ranged from <2 mg kg^−1^ up to 840 mg kg^−1^ [51]. Similarly, putrescine concentrations have been found to range between 0.11–830 mg kg^−1^ and cadaverine concentrations between 0.21–2035 mg kg^−1^ in fish and fishery products [4,31,34,50]. Limited data are available for spermidine and spermine levels in fish products, which are reported to range between 1.38–19.83 mg kg^−1^ for spermidine and 0.85–11.90 mg kg^−1^ for spermine [31,34]. 

## 4. Conclusions

The present work reports the successful use of 1-pyrenesulfonyl chloride (PSCl), a UV excitable amino-reactive fluorophore, as derivatization agent for the HPLC-UV-FLD determination of the biogenic polyamines, putrescine, cadaverine, histamine, spermidine and spermine in fish products. The optimized derivatization procedure resulted in the complete reaction of all the amino-groups, as was confirmed by the LC-ESI-MS spectra. The existence of two or more pyrene moieties in the structure of the derivatives and their favorable conformation induced intramolecular excitement (excimer fluorescence), and, therefore, the shifting of the emission to a longer wavelength. Biogenic amines were detected in all the tested samples of canned fish food, sea bass, anchovy, mackerel, sardines and tuna, collected from the Greek market but there were in all cases below the regulated limit of histamine 100 mg kg^−1^.

## Figures and Tables

**Table 1:** Chromatographic parameters with fluorescence detection of spiked sea bass sample.

	PUT	CAD	HIS c	SPD	SPM
**Retention Time (min)**	22.7	24.1	24.6	30.6	33.7
**Width at 50% of Peak Height (min)**	0.25	0.23	0.19	0.15	0.21
**Asymmetry Factor**	0.92	0.95	0.98	0.87	0.88
**Theoretical Plates (N) ×103**	45	61	94	231	143
**Resolution**	1.7	1.7	2.0	2.5	2.4

**Table 2:** Identified *m/z* ions of the pyrene derivatives of BAs and internal standard (I.S.) by LC-ESI-MS.

Pyrene Derivatives	MW (g/mol)	[M + H]^+^	[M+Na]^+^	[2M+Na]^+^	No of Pyrene Moieties
Putrescine	615.8	617.8	639.8	1256.5	2
Cadaverine	630.9	631.9	653.9	1284.6	2
Histamine	639.8	640.8	662.8	-	2
1,7-diaminoheptane (I.S)	658.9	659.8	681.8	1340.6	2
Spermidine	938.2	-	961.2	-	3
Spermine	1259.5	1260.5	-	-	4

**Table 3:** Standard calibration curves, instrumental limit of detection (LOD_instr_) and quantification (LOQ_instr_) of the derivatized BAs with UV and Fluorescence detectors C: μg mL^−^^1^. y: analyte peak area. Concentration range: 0.1–10 μg mL^−^^1^.

		Calibration Curve	R	LOD_instr_ (μg mL^−^^1^)	LOQ_instr_ (μg mL^−^^1^)
**PUT**	UV	y = (352.6 ± 6.9) × C + (32 ± 30)	0.998	0.03	0.09
	FLD	y = (2148 ± 111) × C + (111 ± 114)	0.992	0.03	0.09
**CAD**	UV	y = (315.8 ± 3.0) × C + (9 ± 13)	0.9995	0.02	0.07
	FLD	y = (1350 ± 55) × C + (240 ± 125)	0.993	0.02	0.06
**SPD**	UV	y = (221.0 ± 8.4) × C + (38 ± 36)	0.993	0.04	0.10
	FLD	y = (1178 ± 45) × C + (35 ± 46)	0.996	0.03	0.10
**SPM**	UV	y = (168.7 ± 4.8) × C + (29 ± 21)	0.996	0.07	0.20
	FLD	y = (959 ± 14) × C + (-21 ± 31)	0.9992	0.05	0.15
**HIS**	UV	y = (180.1 ± 3.6) × C + (30 ± 16)	0.998	0.08	0.20
	FLD	y = (352 ± 16) × C + (37 ± 35)	0.992	0.10	0.30

**Table 4:** Matrix matched calibration curves of sea bass sample, limit of detection (LOD) and quantification (LOQ) of the BAs with UV and Fluorescence detectors C: μg mL^−^^1^. y: analyte peak area. BAs concentration range: 0.2–32 mg kg^−1^ sample, which corresponds to 0.05–8.0 μg mL^−^^1^ measured solution.

		Calibration Curve	R	LOD (mg kg^−^^1^)	LOQ (mg kg^−^^1^)
PUT	UV	y = (239.7 ± 3.3) × C + (-9.6 ± 3.3)	0.9992	0.2	0.6
	FLD	y = (1458 ± 22) × C + (-67 ± 22)	0.9991	0.2	0.6
CAD	UV	y = (237.4 ± 2.0) × C + (1.7 ± 2.0)	0.9997	0.1	0.3
	FLD	y = (1286 ± 16) × C + (-11 ± 16)	0.9994	0.2	0.5
SPD	UV	y = (58.6 ± 2.4) × C + (-0.8 ± 2.4)	0.993	0.5	1.6
	FLD	y = (301 ± 10) × C + (-18 ± 10)	0.995	0.4	1.3
SPM	UV	y = (20.51 ± 0.82) × C + (1.99 ± 0.81)	0.994	0.5	1.6
	FLD	y = (113.4 ± 4.6) × C + (16.3 ± 4.5)	0.994	0.5	1.6
HIS	UV	y = (118.1 ± 5.2) × C + (-20.0 ± 6.3)	0.996	0.7	2.1
	FLD	y = (134.3 ± 8.4) × C + (-46.8 ± 10.1)	0.992	1.0	3.1

**Table 5:** Limit of detection (LOD) for the different matrices.

LODs (mg kg^−1^)	PUT		CAD		SPD		SPM		HIS	
	UV	FLD	UV	FLD	UV	FLD	UV	FLD	UV	FLD
**Tuna**	0.3	0.3	0.4	0.3	0.7	0.5	0.6	1.0	0.7	0.6
**Mackerel**	1.0	1.4	0.5	0.3	0.6	0.6	1.2	0.5	0.2	0.7
**Anchovy**	0.6	0.8	0.5	1.0	0.5	0.5	0.5	0.6	0.5	0.1
**Anchovy Marinated**	0.6	0.7	0.8	0.7	1.0	0.5	1.1	0.5	0.8	0.5
**Sea bass**	0.2	0.2	0.1	0.2	0.5	0.4	0.5	0.5	0.7	1.0
**Sardines**	0.6	0.7	0.6	0.8	0.3	0.2	0.4	1.1	0.2	0.4

**Table 6:** Instrumental and method precision data under intra and inter day conditions with UV and FLD detectors, with analytical parameter the Peak Area (PA) and the ratio *analyte peak area/internal standard peak area* (F). BAs concentrations in tested sea bass sample for the method intra and inter-day assays: 2–10 mg kg^−1^.

	Instrumental Precision 0.5 μg mL^−1^, %RSD, *n* = 5				Method Intra-Day Assay %RSD, *n* = 6				Method Inter-Day Assay %RSD, *n* = 6, k = 2			
	UV	UV	FLD	FLD	UV	UV	FLD	FLD	UV	UV	FLD	FLD
	PA	F	PA	F	PA	F	PA	F	PA	F	PA	F
**PUT**	2.3	0.1	2.6	0.4	8.2	9.3	10	8.2	8.9	12	9.6	15
**CAD**	2.3	0.3	2.7	0.5	8.2	9.1	7.4	6.1	9.5	12	11	16
**SPD**	2.4	0.3	2.7	0.4	12	8.7	12	8.8	14	13	21	15
**SPM**	1.8	0.7	2.8	0.5	13	15	14	11	12	19	14	23
**HIS**	4.2	3.9	4.2	2.3	10	9.4	11	12	11	9.4	10	14

**Table 7:** Absolute and relative recoveries of each compound from spiked sea bass sample at six different levels (1.6, 3.2, 8.0, 12.8, 16.0 and 32 mg kg^−1^), *n* = 6. Effect of standard addition.

	UV			FLD		
Compound	Absolute Recovery (%)	Relative Recovery % (±SD)		Absolute Recovery (%)	Relative Recovery % (±SD)	
**PUT**	68	88	(±12)	68	86	(±15)
**CAD**	75	102	(±2.6)	95	98	(±2.1)
**SPD**	27	98	(±12)	26	88	(±6.6)
**SPM**	12	112	(±8.0)	12	114	(±6.5)
**HIS**	66	72	(±13)	38	67	(±11)

**Table 8:** Βiogenic amines’ concentrations (mg kg^−1^) in canned fish samples.

Content of BAs (mg kg^−1^)	PUT	CAD	SPD	SPM	HIS	Sum of BAs
**Tuna**	ND	<0.9	2.4	7.6	ND	10.9
**Mackerel**	40.1	0.9	3.6	2.5	2.5	49.6
**Anchovy**	2.4	3.0	<1.5	<1.8	5.8	14.5
**Anchovy Marinated**	3.8	5.6	<1.5	<1.5	<1.5	13.9
**Sea bass**	<0.6	ND	1.4	2.1	ND	4.1
**Sardines**	2.7	10.8	8.1	9.6	3.7	34.9

ND: Not Detected (below the limit of detection). “<”: Detected but below the limit of quantification.

## Supplementary Materials

The following are available online at https://www.mdpi.com/2304-8158/9/5/609/s1, Figure S1: (A) HPLC-FLD; (B) HPLC-UV chromatogram of sea bass tissue, fortified only with the internal standard 1,7 diaminoheptane (IS) at 4.8 mg kg^−1^, Figure S2: Reconstructed Ion Chromatograms of a 5 μg mL^−1^ histamine pyrene derivative at *m*/*z* 640.3–641.3 [M+H]+ and 662.3–653.3 [M+Na]+, Figure S3: Full scan spectrum (*m*/*z* 500-1500) at the retention time of cadaverine pyrene derivative (8.4 min) of a 5 μg mL^−1^ standard solution, Figure S4: Reconstructed Ion Chromatograms of a 5 μg mL^−1^ cadaverine pyrene derivative at *m*/*z* 631.3–632.3 [M+H]+, 652.3–653.3 [M+Na]+ and 1283.4–1284.4 [2M+Na]+, Figure S5: Matrix matched calibration curves of sea bass sample with Fluorescence detector.

## References

[1] Baixas-Nogueras S., Bover-Cid S., Veciana-Nogués M.T., Mariné-Font A., Vidal-Carou M.C. (2005). Biogenic Amine Index for Freshness Evaluation in Iced Mediterranean Hake (Merluccius merluccius). J. Food Prot..

[2] Ruiz-Capillas C., Herrero A.M. (2019). Impact of Biogenic Amines on Food Quality and Safety. Foods.

[3] Doeun D., Davaatseren M., Chung M. (2017). Biogenic amines in foods. Food Sci. Biotechnol..

[4] European Food Safety Authority (2011). Scientific Opinion on risk based control of biogenic amine formation in fermented foods. EFSA J..

[5] Mietz J.L. (1977). Chemical quality index of canned tuna as determined by high-pressure liquid chromatography. J. Food Sci..

[6] Shalaby A.R. (1996). Significance of biogenic amines to food safety and human health. Food Res. Int..

[7] Bulushi I.A., Poole S., Deeth H.C., Dykes G.A. (2009). Biogenic Amines in Fish: Roles in Intoxication, Spoilage, and Nitrosamine Formation—A Review. Crit. Rev. Food Sci. Nutr..

[8] (2005). European Commission. Commission Regulation (EC) No 2073/2005 of 15 November 2005 on microbiological criteria for foodstuffs. Off. J. Eur. Union.

[9] Fish and Fishery Products Hazards and Controls Guidance. https://www.fda.gov/Food/GuidanceRegulation/GuidanceDocumentsRegulatoryInformation/Seafood/ucm2018426.htm.

[10] Zhang N., Wang H., Zhang Z.X., Deng Y.H., Zhang H.S. (2008). Sensitive determination of biogenic amines by capillary electrophoresis with a new fluorogenic reagent 3-(4-fluorobenzoyl)-2-quinolinecarboxaldehyde. Talanta.

[11] An D., Chen Z., Zheng J., Chen S., Wang L., Huang Z., Weng L. (2015). Determination of biogenic amines in oysters by capillary electrophoresis coupled with electrochemiluminescence. Food Chem..

[12] Daniel D., Dos Santos V.B., Vidal D.T., do Lago C.L. (2015). Determination of biogenic amines in beer and wine by capillary electrophoresis-tandem mass spectrometry. J. Chromatogr. A.

[13] Plotka-Wasylka J., Simeonov V., Namiesnik J. (2016). An in situ derivatization - dispersive liquid-liquid microextraction combined with gas-chromatography - mass spectrometry for determining biogenic amines in home-made fermented alcoholic drinks. J. Chromatogr. A.

[14] Cunha S.C., Faria M.A., Fernandes J.O. (2011). Gas chromatography-mass spectrometry assessment of amines in Port wine and grape juice after fast chloroformate extraction/derivatization. J. Agric. Food Chem..

[15] De Borba B.M., Rohrer J.S. (2007). Determination of biogenic amines in alcoholic beverages by ion chromatography with suppressed conductivity detection and integrated pulsed amperometric detection. J. Chromatogr. A.

[16] Tao Z., Sato M., Han Y., Tan Z., Yamaguchi T., Nakano T. (2011). A simple and rapid method for histamine analysis in fish and fishery products by TLC determination. Food Control..

[17] Dadáková E., Křížek M., Pelikánová T. (2009). Determination of biogenic amines in foods using ultra-performance liquid chromatography (UPLC). Food Chem..

[18] Costa M.P., Balthazar C.F., Rodrigues B.L., Lazaro C.A., Silva A.C., Cruz A.G., Conte Junior C.A. (2015). Determination of biogenic amines by high-performance liquid chromatography (HPLC-DAD) in probiotic cow’s and goat’s fermented milks and acceptance. Food Sci. Nutr..

[19] Liu S.J., Xu J.J., Ma C.L., Guo C.F. (2018). A comparative analysis of derivatization strategies for the determination of biogenic amines in sausage and cheese by HPLC. Food Chem..

[20] Wang Y.Q., Ye D.Q., Zhu B.Q., Wu G.F., Duan C.Q. (2014). Rapid HPLC analysis of amino acids and biogenic amines in wines during fermentation and evaluation of matrix effect. Food Chem..

[21] Mayer H.K., Fiechter G., Fischer E. (2010). A new ultra-pressure liquid chromatography method for the determination of biogenic amines in cheese. J. Chromatogr. A.

[22] Lázaro C.A., Conte-Júnior C.A., Cunha F.L., Mársico E.T., Mano S.B., Franco R.M. (2013). Validation of an HPLC Methodology for the Identification and Quantification of Biogenic Amines in Chicken Meat. Food Anal. Method.

[23] Jia S., Ryu Y., Kwon S.W., Lee J. (2013). An in situ benzoylation-dispersive liquid-liquid microextraction method based on solidification of floating organic droplets for determination of biogenic amines by liquid chromatography-ultraviolet analysis. J. Chromatogr. A.

[24] Redruello B., Ladero V., Cuesta I., Alvarez-Buylla J.R., Martin M.C., Fernandez M., Alvarez M.A. (2013). A fast, reliable, ultra high performance liquid chromatography method for the simultaneous determination of amino acids, biogenic amines and ammonium ions in cheese, using diethyl ethoxymethylenemalonate as a derivatising agent. Food Chem..

[25] De Mey E., Drabik-Markiewicz G., De Maere H., Peeters M.C., Derdelinckx G., Paelinck H., Kowalska T. (2012). Dabsyl derivatisation as an alternative for dansylation in the detection of biogenic amines in fermented meat products by reversed phase high performance liquid chromatography. Food Chem..

[26] Kang L., You J., Sun Z., Wang C., Ji Z., Gao Y., Suo Y., Li Y. (2011). LC Determination of Trace Biogenic Amines in Foods Samples with Fluorescence Detection and MS Identification. Chromatographia.

[27] Tahmouzi S., Khaksar R., Ghasemlou M. (2011). Development and validation of an HPLC-FLD method for rapid determination of histamine in skipjack tuna fish (Katsuwonus pelamis). Food Chem..

[28] Wu H., Li G., Liu S., Ji Z., Zhang Q., Hu N., Suo Y., You J. (2014). Simultaneous Determination of Seven Biogenic Amines in Foodstuff Samples Using One-Step Fluorescence Labeling and Dispersive Liquid–Liquid Microextraction Followed by HPLC-FLD and Method Optimization Using Response Surface Methodology. Food Anal. Method.

[29] Triki M., Jiménez-Colmenero F., Herrero A.M., Ruiz-Capillas C. (2012). Optimisation of a chromatographic procedure for determining biogenic amine concentrations in meat and meat products employing a cation-exchange column with a post-column system. Food Chem..

[30] Kelly M.T., Blaise A., Larroque M. (2010). Rapid automated high performance liquid chromatography method for simultaneous determination of amino acids and biogenic amines in wine, fruit and honey. J. Chromatogr. A.

[31] Sánchez J.A., Ruiz-Capillas C. (2011). Application of the simplex method for optimization of chromatographic analysis of biogenic amines in fish. Eur. Food Res. Technol..

[32] Zhao Q.X., Xu J., Xue C.H., Sheng W.J., Gao R.C., Xue Y., Li Z.J. (2007). Determination of Biogenic Amines in Squid and White Prawn by High-Performance Liquid Chromatography with Postcolumn Derivatization. J. Agric. Food Chem..

[33] Zotou A., Notou M. (2012). Enhancing Fluorescence LC Analysis of Biogenic Amines in Fish Tissues by Precolumn Derivatization with Naphthalene-2,3-dicarboxaldehyde. Food Anal. Method.

[34] Li G., Dong L., Wang A., Wang W., Hu N., You J. (2014). Simultaneous determination of biogenic amines and estrogens in foodstuff by an improved HPLC method combining with fluorescence labeling. LWT Food Sci. Technol..

[35] Marks Rupp H.S., Anderson C.R. (2005). Determination of putrescine and cadaverine in seafood (finfish and shellfish) by liquid chromatography using pyrene excimer fluorescence. J. Chromatogr. A.

[36] Lozanov V., Petrov S., Mitev V. (2004). Simultaneous analysis of amino acid and biogenic polyamines by high-performance liquid chromatography after pre-column derivatization with N-(9-fluorenylmethoxycarbonyloxy)succinimide. J. Chromatogr. A.

[37] Papageorgiou M., Lambropoulou D., Morrison C., Kłodzińska E., Namieśnik J., Płotka-Wasylka J. (2018). Literature update of analytical methods for biogenic amines determination in food and beverages. TrAC Trends Anal. Chem..

[38] Ochi N. (2019). Simultaneous determination of eight underivatized biogenic amines in salted mackerel fillet by ion-pair solid-phase extraction and volatile ion-pair reversed-phase liquid chromatography-tandem mass spectrometry. J. Chromatogr. A.

[39] Kaufmann A., Maden K. (2018). Easy and Fast Method for the Determination of Biogenic Amines in Fish and Fish Products with Liquid Chromatography Coupled to Orbitrap Tandem Mass Spectrometry. J. AOAC Int..

[40] Zhang Y.J., Zhang Y., Zhou Y., Li G.H., Yang W.Z., Feng X.S. (2019). A review of pretreatment and analytical methods of biogenic amines in food and biological samples since 2010. J. Chromatogr. A.

[41] Nishikawa H., Tabata T., Kitani S. (2012). Simple Detection Method of Biogenic Amines in Decomposed Fish by Intramolecular Excimer Fluorescence. Food Nutr. Sci..

[42] Nohta H., Satozono H., Koiso K., Yoshida H., Ishida J., Yamaguchi M. (2000). Highly Selective Fluorometric Determination of Polyamines Based on Intramolecular Excimer-Forming Derivatization with a Pyrene Labeling Reagent. Anal. Chem..

[43] Yoshitake T., Ichinose F., Yoshida H., Todoroki K., Kehr J., Inoue O., Nohta H., Yamaguchi M. (2003). A sensitive and selective determination method of histamine by HPLC with intramolecular excimer-forming derivatization and fluorescence detection. Biomed. Chromatogr..

[44] Nakano T., Todoroki K., Ishii Y., Miyauchi C., Palee A., Min J.Z., Inoue K., Suzuki K., Toyo’oka T. (2015). An easy-to-use excimer fluorescence derivatization reagent, 2-chloro-4-methoxy-6-(4-(pyren-4-yl)butoxy)-1,3,5-triazine, for use in the highly sensitive and selective liquid chromatography analysis of histamine in Japanese soy sauces. Anal. Chim. Acta.

[45] DeSilva K.H., Vest F.B., Kames H.T. (1996). Pyrene Sulphonyl Chloride as a Reagent for Quantitation of Oestrogens in Human Serum Using HPLC with Conventional and Laser-Induced Fluorescence Detection. Biomed. Chromatogr..

[46] Paiva B.T., Tominaga M., Paiva M.C. (1970). Ionization of Histamine, N-Acetylhistamine, and Their Iodinated Derivatives. J. Med. Chem..

[47] PubChem. https://pubchem.ncbi.nlm.nih.gov/compound/Cadaverine#section=Dissociation-Constants.

[48] PubChem. https://pubchem.ncbi.nlm.nih.gov/compound/1_4-Diaminobutane#section=pKa.

[49] DRUGBANK. https://www.drugbank.ca/drugs/DB03566.

[50] DRUGBANK. https://www.drugbank.ca/drugs/DB00127.

[51] Ly D., Mayrhofer S., Schmidt J.-M., Zitz U., Domig K.J. (2020). Biogenic Amine Contents and Microbial Characteristics of Cambodian Fermented. Foods.

