# Supplementary material for: Liquid Chromatographic Determination of Biogenic Amines in Fish Based on Pyrene Sulfonyl Chloride Pre-Column Derivatization
Source: Foods. 2020 May 9;9(5):609. doi: 10.3390/foods9050609 (PMC7278825; doi:10.3390/foods9050609)
Supplement: Supplementary file 1 [file foods-09-00609-s001.pdf]

Supplementary Material

Liquid Chromatographic Determination of Biogenic Amines in Fish based on Pyrene Sulfonyl Chloride Pre-Column Derivatization

Elvira S. Plakidi [1, a], Niki C. Maragou [1,*,b] , Marilena E. Dasenaki [1], Nikolaos C. Megoulas [1, c], Michael A. Koupparis [1] and Nikolaos S. Thomaidis [1]

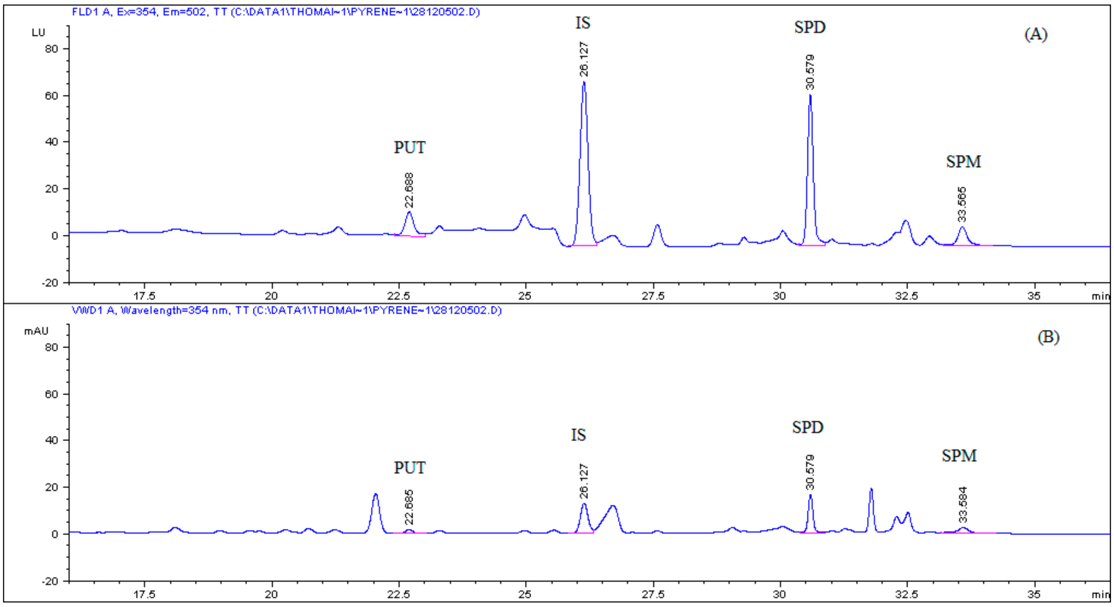

Figure S1. (A) HPLC-FLD; (B) HPLC-UV chromatogram of sea bass tissue, fortified only with the internal standard 1,7 diaminoheptane (IS) at 4.8 mg kg-1.

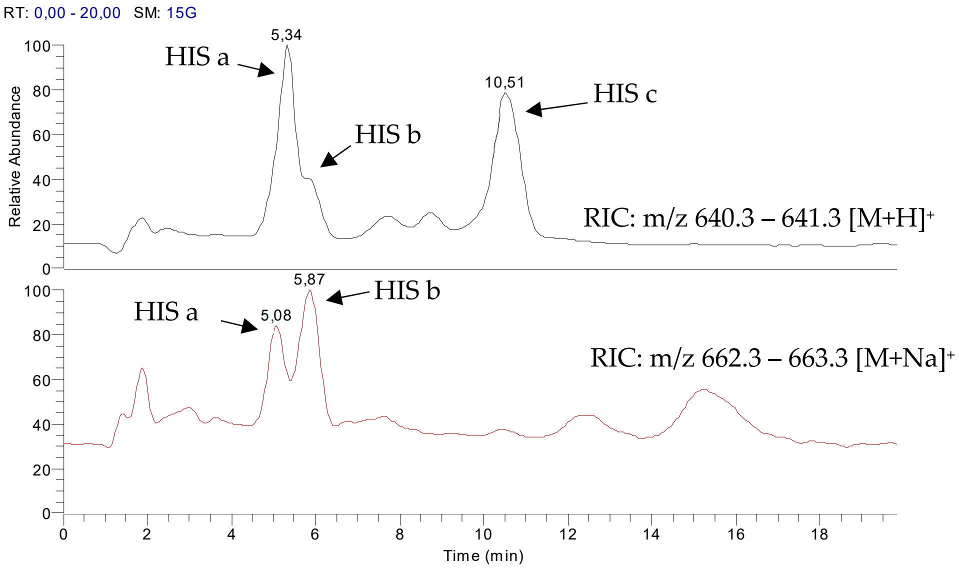

Figure 2. Reconstructed Ion Chromatograms of a 5 μg mL^{-1} histamine pyrene derivative at m/z 640.3 – 641.3 [M+H]$^+$ and 662.3 – 653.3 [M+Na]$^+$.

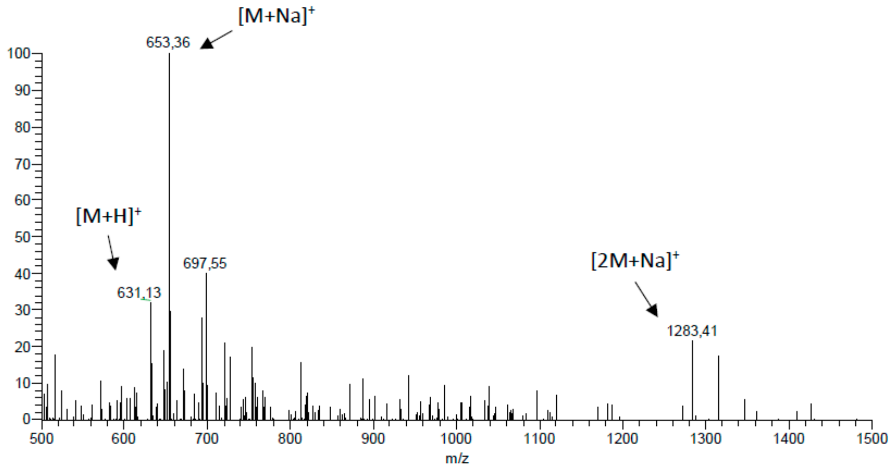

Figure 3. Full scan spectrum (m/z 500-1500) at the retention time of cadaverine pyrene derivative (8.4 min) of a 5 μg mL^{-1} standard solution.

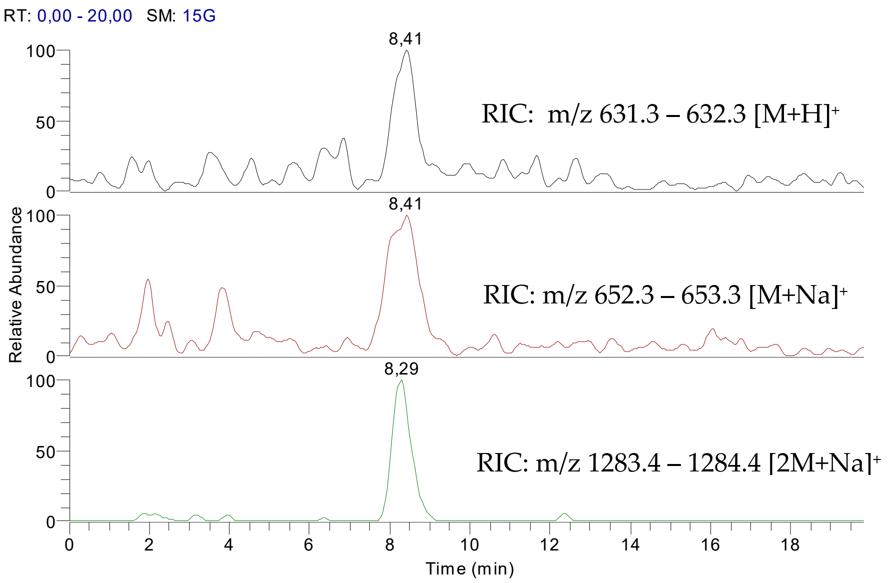

Figure S4. Reconstructed Ion Chromatograms of a 5 μg mL^{-1} cadaverine pyrene derivative at m/z 631.3 – 632.3 $[M+H]^+$, 652.3 – 653.3 $[M+Na]^+$ and 1283.4 – 1284.4 $[2M+Na]^+$.

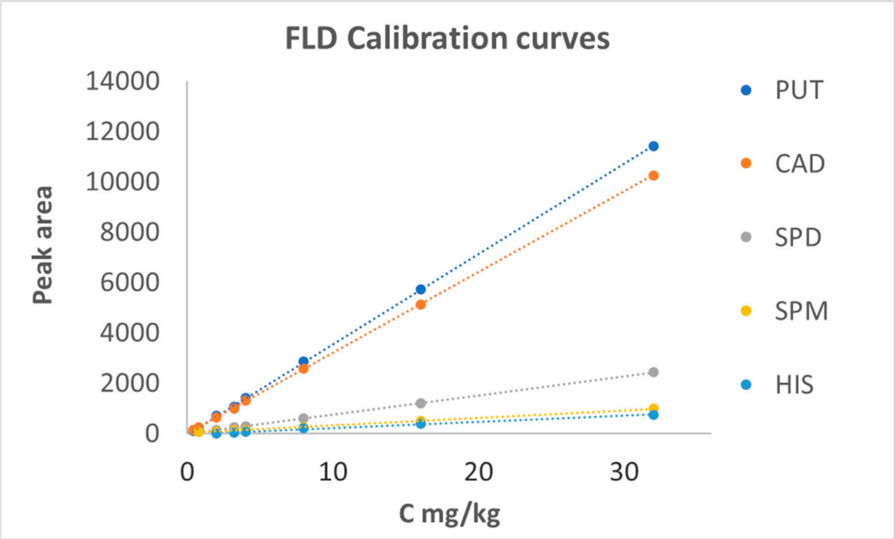

Figure 5. Matrix matched calibration curves of sea bass sample with Fluorescence detector.
